# Diabetic pregnancy: A literature review of maternal and neonatal adverse outcomes

**DOI:** 10.18502/ijrm.v23i2.18482

**Published:** 2025-05-01

**Authors:** Sara Mohamed, Waverly Kundysek, Niraj Vora, Vinayak Govande, Raza Bajwa, Mohammad Nasir Uddin

**Affiliations:** ^1^Department of Neonatology, Baylor Scott & White Health, Temple, Texas, USA.; ^2^Texas A&M University School of Medicine, College Station, Texas, USA.

**Keywords:** Diabetes mellitus, Pregnancy, Maternal health, Pre-eclampsia, Neonatal health.

## Abstract

Of all pregnant women in the United States an average of 1.5% reported to have type 1 or type 2 diabetes mellitus. Our review article will discuss and explore the relationship between pre-pregnancy diabetes and its adverse outcomes in mothers and neonates. Diabetes in pregnancy can cause a myriad of complications, many of which are related to microvascular changes, including diabetic nephropathy and retinopathy associated with preterm delivery, cesarean sections, and intrauterine growth restriction. Pregnant patients with diabetes also have an increased risk of pre-eclampsia, likely due to complications related to abnormal structure and function of the placenta. In addition, cardiovascular complications are more common and may present antepartum, intrapartum, or postpartum. Adverse neonatal outcomes that have been observed in diabetic pregnancies include fetal stillbirth and perinatal death, macrosomia, congenital malformations, respiratory distress, and neurological impairments. These complications explain the increased morbidity and mortality rate of infants of diabetic mothers, and the increased frequency of neonatal intensive care unit hospitalizations after birth. Diabetes in pregnancy causes a spectrum of changes in the maternal-fetal interface. This review addresses the placental changes during pregnancy and its adverse maternal and neonatal outcomes. We strongly believe the material discussed in this article can help in understanding the effects of diabetes during pregnancy which will ultimately aid in designing interventions to prevent these adverse outcomes.

## 1. Introduction

The term diabetes mellitus describes the disease of abnormal carbohydrate metabolism. Hyperglycemia usually results from varying peripheral resistance to insulin action, defective insulin secretion, and disease complications. Abnormally high glucose levels during pregnancy can occur due to non-gestational diabetes mellitus. It is known that first-trimester glycosylated hemoglobin levels > 7.0% contribute to the worst outcomes (1).

More recently, it has been proved that hyperglycemia can contribute to abnormal invasive and proliferative cytotrophoblastic (CTB) placental cell development (2). During the early phase of pregnancy (from conception to 12 wk), CTB cells are believed to have various functions to facilitate the invasion of the maternal host, this is essential to ensure appropriate maternal blood transfer to the placenta (3). The mitogen-activated protein kinase is directly involved in CTB growth, differentiation, invasiveness, and programmed cell death (4–9) and the interleukin-6 can indirectly affect the previous process (9), this is all prominent during stress on a cellular level. Peroxisome proliferator-activated receptor gamma (PPAR-γ) induces the proliferation and differentiation of the villi of the trophoblasts. Multiple studies have established the effect of PPAR-γ on the stimulation of villous trophoblasts and endocrine function (10–12). Glucose can change the mechanism of the CTB invasion and its dispersion via stress signaling which is mainly controlled by p38 and mitogen-activated protein kinase pathways (13).

The vascular process at the placental interface involves multiple regulatory pathways of angiogenic factors; vascular endothelial growth factor and placental growth factor, and anti-angiogenic markers; soluble fms-like tyrosine kinase-1 and soluble endoglin (14–18). These markers are believed to be altered when exposed to impaired higher glucose levels, creating an anti-angiogenic profile. Hyperglycemia impairs the invasive and proliferative profile of first-trimester CTB cells (18).

The aim of this literature review is to explore the relationship between diabetic pregnancy and its adverse outcomes in mothers and neonates.

**Figure 1:**
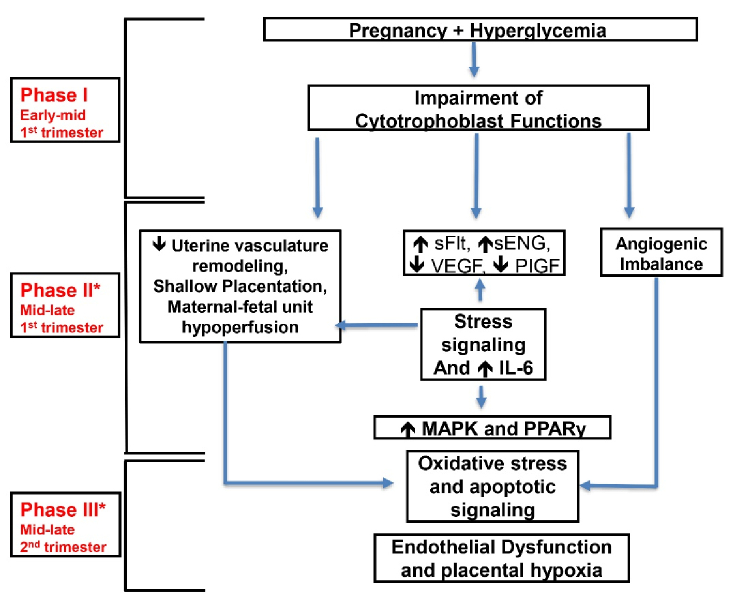
Schematic diagram of the phenomena depicted in hyperglycemic effects on the placenta during pregnancy's first and second trimester. Hyperglycemia triggers dysfunction in cytotrophoblasts, leading to the activation of stress signaling pathways. This cascade of events results in hypoperfusion of the maternal-fetal unit and apoptotic changes. Key factors involved in this process include. sFlt: Soluble fms-like tyrosine kinase, sENG: Soluble endoglin, VEGF: Vascular endothelial growth factor, MAPK: Mitogen-activated protein kinase, PPAR: Peroxisome proliferator-activated receptor, PIGF: Placental growth factor, IL-6: Interleukin-6.

These complications can result in adverse effects for both the mother and infant. This phenomenon has been depicted in figure 1.

## 2. Maternal Outcomes in Pregnancies Complicated with Diabetes

Maternal outcomes are commonly observed in hospital setups with diabetic pregnancies.

### Preterm delivery and low birth weight 

The most significant diabetic complications reported are nephropathy and retinopathy. The effect of diabetes on the vascular renal system is the highest compared to the rest of the vascular complications. This can explain the significant effects on outcomes perinatally. The literature review revealed that nephropathy was significantly related to the incidence of preterm delivery and very low birth weight. The other 3 factors (pre-eclampsia, intrauterine growth restriction, and fetal distress) are all common sequelae and/or associated with nephropathy due to their strong association with chronic hypertension (19).

### Mode of delivery

The cesarean section rate is higher in diabetic pregnancies compared to non-diabetes mellitus (DM) pregnancies and it is higher in preterm deliveries compared to term ones (20, 21). The main indications for a preterm cesarean section are related to fetal distress and diabetic complications, whereas the main indication for an at-term cesarean section is cephalopelvic disproportion (22).

### Pre-eclampsia 

Women with pregestational diabetes (type 1 DM, type 2 DM) and gestational diabetes continue to have poorer pregnancy outcomes than the background population (23, 24). The risk of pre-eclampsia is increased in women with diabetes; however, the exact mechanism of pre-eclampsia is not entirely understood.

Multiple factors including endocrine pathways and neurohormonal pathways are altered which contributes to the course of pre-eclampsia. This is also believed to be exacerbated by uncontrolled diabetes status. It is believed that the main contributors to the mechanism of pre-eclampsia are the imbalance in angiogenesis, cardiotonic steroids, and autoantibodies that target angiotensin receptor type 1. All of the previous can be exacerbated by cardiac targeting steroids, genetic liability, and immunological susceptibility, hence the presence of vascular compromise (25–28).

### Cardiovascular complications

Analysis of pooled studies has revealed that pregnant individuals diagnosed with DM exhibit a doubled chance of cardiovascular events postpartum when compared to their counterparts (29). Recognizing the significance of this risk, the American Heart Association incorporates a history of diabetes into its cardiovascular risk factor classification for women (28, 29).

### Placental complications

Hyperglycemia impacts the ability of CTB cells to invade during placental formation. Typically, CTB cell invasion is facilitated by converting plasminogen to plasmin through the action of a urokinase plasminogen (uPA) activator. The activity of uPA is controlled by plasminogen activator inhibitor-1; both plasminogen activator inhibitor-1 and uPA are downregulated at the level of CTB cells subjected to hyperglycemic insults. Vascular endothelial growth factor and placental growth factor, essential pro-angiogenic factors for placental development, experience downregulation, while antiangiogenic factors such as soluble fms-like tyrosine kinase-1 and soluble endoglin, which hinder normal placentation, can be upregulated. This hyperglycemia-induced dysregulation activates stress signaling pathways, leading to aberrant placentation, potentially resulting in complications like pre-eclampsia during later stages of pregnancy (11).

### Number of days of hospitalizations

In a study completed in Australia, rates of placenta previa, placental abruption, postpartum hemorrhage, and antepartum hemorrhage were similar in diabetic vs. nondiabetic women. Despite the similarity in risk comorbidities, the rate of intensive care unit admission was 1.9% in women with diabetes compared to 0.2% in women with no diabetes (30). Table I summarizes placental pathophysiology and adverse outcomes in diabetic pregnancy.

**Table 1:** Different levels of glucose in pregestational and gestational diabetes and its effect on the placental tissue

**Patient type**	**Pregestation**	**Pregnancy**		**Placenta**	**Risk of pre-eclampsia**
		**0–20 wk of gestation**	**20–40 wk of gestation**		
**Normal**	Normal	Normal	Normal	Normal	5–8%
**Gestational diabetes**	Normal	Normal	Elevated	Increased	Up to 10%
**Pregestational diabetes**	Elevated	Elevated	Elevated	Decreased	Up to 22%

## 3. Neonatal Outcomes in Diabetic Pregnancies

Diabetic pregnancy can contribute to multiple neonatal outcomes.

### Stillbirth as well as perinatal death

There are reports from multiple studies that show an increased incidence of stillbirth with DM (31, 32). The main causes of stillbirth in the setting of DM are associated with abnormal placentation process leading to congenital malformations and intrauterine growth restriction (33). On the fetal side, hyperglycemia can lead to anaerobic metabolism with hypoxia resulting in acidosis, which is believed to be a contributing factor to stillbirth (34). The previous findings emphasize the importance of tight management of diabetes to minimize the morbidities and comorbidities associated with poor glycemic control.

### Fetal growth and macrosomia

It is important to note that the neonatal morbidity and mortality rate is higher in infants of diabetic mothers. Neonatal intensive care unit admissions are higher for neonates of diabetic mothers for many different reasons, as detailed below (1).

Untreated diabetes during pregnancy is frequently linked to fetal macrosomia, defined as children with birth weight greater than 4000 gr as shown by multiple studies (35, 36). The placenta mainly controls the state of transient insulin resistance during pregnancy, the transient resistance is believed to normalize after birth. The placenta secretes hormones, adipokines, and cytokines to maternal circulation that can induce insulin resistance (37). The normal hormonal regulatory pathways can be opposed via human placental lactogen, human placental growth hormone, and human chorionic gonadotrophin (38, 39). Growth hormone and placental lactogen increase maternal insulin-like growth factor (IGF) and metabolic gluconeogenesis and lipolysis. It is believed that the primary regulator of maternal IGF is the placental growth hormone (40). The placenta secretes elevated levels of IGF-I, IGF-II, IGF-IR, and IGF-IIR mRNA, which are associated with fetal macrosomia (41). Furthermore, maternal insulin resistance increases the amount of glucose passing through the placenta to the infant, this is believed to be the major reason for macrosomia (excess glucose is stored as body fat) in DM during pregnancy (42, 43). Macrosomia was associated with a higher chance of shoulder dystocia and neonatal hypoglycemia (44).

### Congenital malformations

Congenital malformations are a significant concern in diabetic pregnancies. Studies show that the risk of congenital anomalies in infants born to diabetic mothers can be as high as 10%, compared to 2–5% in infants of mothers with normal blood sugar levels (45, 46). The risk is even greater when first-trimester glycosylated hemoglobin levels are elevated (44). Among these malformations, heart defects are the most common, accounting for up to 40% of all anomalies. Other systems, like the extremities, neural tube, and musculoskeletal systems, are also frequently affected (16, 47). The typical cardiac issues include atrioventricular septal defects, hypoplastic left heart syndrome, and persistent truncus arteriosus (15, 23, 25, 26). Diabetes during pregnancy has also been linked to higher resting fetal heart rates early in pregnancy (27) and an increased risk of conditions like sacral agenesis, holoprosencephaly, spina bifida, and cleft lip or palate (47). These findings require careful monitoring and management of diabetes in pregnancy.

### Respiratory distress

Respiratory distress is directly proportional to poor maternal glycemic control at all gestational stages. In addition, it can hinder fetal pulmonary maturation secondary to fetal hyperglycemia that induces hyperinsulinemia. Fetal hyperglycemia and hyperinsulinemia are suggested mechanisms for delayed pulmonary maturation. At birth, infants may require positive pressure ventilation support or intubation, along with supplemental oxygen (35).

**Figure 2:**
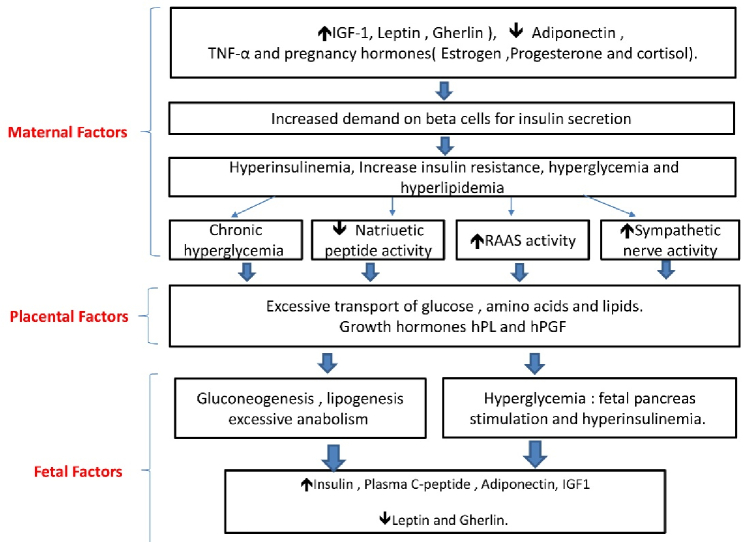
Inflammatory, immunological, and hormonal factors causing systemic and maternal-fetal interface changes. Insulin resistance is augmented by growth hormones secreted by the placenta and then to systemic circulation. Adipose tissue secretes various hormones and cytokines, including leptin, adiponectin, and tumor necrosis factor-alpha (TNF-α), which can contribute to increased insulin resistance. This creates a cascade of events resulting in hyperglycemia and placental changes. The fetus's insulin levels increase in response to maternal hyperglycemia with hyperinsulinemia. IGF-1: Insulin growth factor-1, hPGH: Human placental growth hormone, RAAS: Renin-angiotensin aldosterone system, hPL: Human placental lactogen.

The marker for lung maturation in the amniotic fluid; phosphatidyl-glycerol, is found to be low or delayed, which is positively correlated to maternal glycemic control (48). One study investigated how intra-fetal glucose infusion affected mRNA expression in the lungs of late-gestation sheep fetuses. Although they found no alteration in the quantity of surfactant-secreting pneumocytes, the study indicated a decrease in the expression of surfactant protein mRNA following glucose infusion (49). Studies showed that insulin can affect genetic transcription on a cellular level, which can decrease the amount of surfactant protein in human lung epithelial cells (48–50). The previous mechanisms of delayed lung maturity and the delay in surfactant secretion may explain the increased incidence of respiratory distress syndrome among diabetic pregnancies. This prodigy has been schematically presented in figure 2.

### Neurodevelopmental outcomes

Maternal diabetes causes profound neonatal hypoglycemia, presumably from the starvation of the brain and stress-induced damage to the brain tissues. This pathology can result in poor neurodevelopmental outcomes for neonates born at term and preterm (51). There is an associated risk between neonatal hypoglycemia and an increased risk of neonatal seizures, as well as white matter reduction on term-equivalent MRI scans (3, 51). Flaccid hypotonia with apnea and coma have also been reported (52).

**Figure 3:**
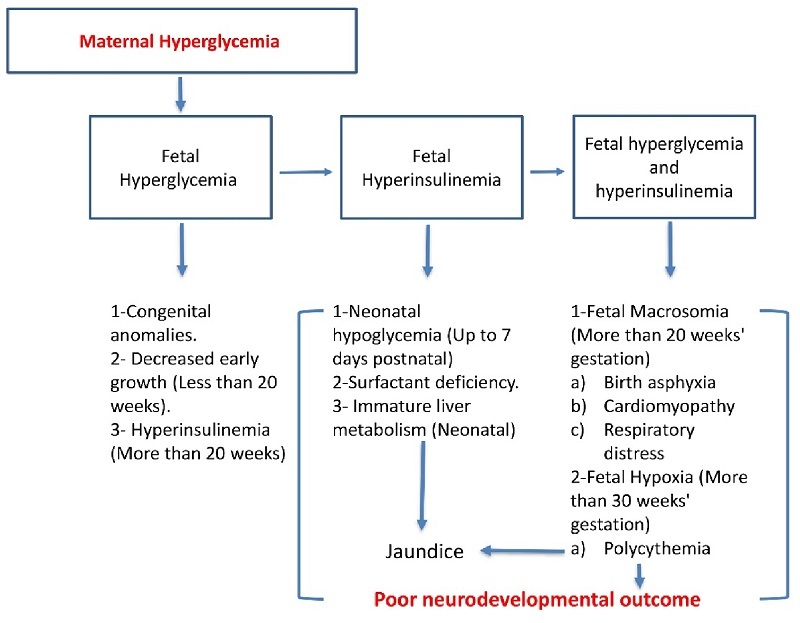
Schematic presentation of the consequences of maternal diabetes causes poor neonatal neurodevelopmental outcomes.

In addition to adverse outcomes during the neonatal period, many other effects on the infant may present later in life. There is an association with delayed brain maturity that results in neuronal and behavioral changes. This contributes to lower intelligence than average babies, language impairments, visual impairments, difficulty in concentration, impulsivity, and cognitive deficits (53, 54). There is an increased risk of schizophrenia and autism spectrum disorders in diabetic pregnancies (55, 56). The neurodevelopmental outcomes have been schematically presented in figure 3.

## 4. Conclusion

The presence of diabetes in pregnancy causes significant risks to both maternal and fetal health. Optimization of diabetes management in pregnancy includes multiple aspects, including glucose regulation, dietary advice, monitoring weight gain during pregnancy, and adequate blood pressure ranges. Careful attention to comorbid conditions by a multidisciplinary team, including maternal-fetal medicine physicians and multiple specialists such as neonatologists, endocrinologists, ophthalmologists, and developmental clinics, can help decrease these risks and ensure the quality of diabetes care before, during, and after pregnancy.

##  Data Availability

Not applicable.

##  Author Contributions

Composing and preparing the article: Conceptualization: M. Nasir Uddin, R. Bajwa, and N. Vora. Writing-original draft preparation, S. Mohamed, W. Kundysek, and V. Govande. Planning, reviewing, and approving of the article for submission: Review and editing, M. Nasir Uddin, R. Bajwa, V. Govande, and N. Vora: Submission, M. Nasir Uddin: Supervision, M. Nasir Uddin.

##  Conflict of Interest

The authors declare that there is no conflict of interest.
